# Canine Adenoviruses in Wildlife: Role in At-Risk Species Conservation and Interface with Domestic Animals

**DOI:** 10.3390/pathogens14020200

**Published:** 2025-02-18

**Authors:** Andrea Balboni, Martina Magliocca, Lorenza Urbani, Mara Battilani

**Affiliations:** Department of Veterinary Medical Sciences, Alma Mater Studiorum-University of Bologna, 40064 Ozzano Emilia, Bologna, Italy; martina.magliocca2@unibo.it (M.M.); lorenza.urbani2@unibo.it (L.U.); mara.battilani@unibo.it (M.B.)

**Keywords:** canine adenovirus type 1, canine adenovirus type 2, diagnostic methods, prevalence, wildlife

## Abstract

Canine adenovirus type 1 (CAdV-1) and type 2 (CAdV-2) are well known pathogens of domestic dogs but are little investigated in wild animals. The few available studies about CAdV-1 in wild animals show that it circulates in various species and that transmission of the virus in the interface between wildlife and domestic animals is a frequent event. Furthermore, wild animals are usually subject to asymptomatic infections, but cases of serious and fatal diseases have been documented, with possible effects on the conservation of the species. In contrast, CAdV-2 infection was reported only recently and sporadically in some wild animals, with few data regarding its pathogenic role in these species. However, the real prevalence of these viruses in wildlife is still uncertain due to the use of serological tests that are largely unable to distinguish antibodies against CAdV-1 and CAdV-2. This review, reporting all the data currently available on CAdV-1 and CAdV-2 infection in wild animals, highlights the importance of these pathogens for wildlife conservation and their role in the potential transmission of the infection to domestic dogs.

## 1. History and General Properties of Canine Adenovirus (CAdV)

There are two types of canine adenovirus (namely *Mastadenovirus canidae* or Canine mastadenovirus A species, CAdV), CAdV type 1 (CAdV-1) and CAdV type 2 (CAdV-2), distinguishable by their genetic, antigenic, and pathogenetic characteristics.

The first report of CAdV-1 infection dates back to 1925, when an epidemic of “fox distemper” was observed in silver fox (a melanistic form of the red fox, *Vulpes vulpes*) ranches in Minnesota, characterized by respiratory, nervous, and intestinal clinical signs associated with a congested liver [1]. Only a few years later, the infection was linked to the virus responsible for epizootic encephalitis in foxes and infectious canine hepatitis (ICH), or Rubarth disease, in dogs [2,3]. However, the virus was isolated for the first time in 1954, and it was attenuated through passages on canine and swine cell lines to produce modified live vaccines (MLV) [4,5]. Other mammalian carnivore species belonging to the *Canidae*, *Mustelidae,* and *Ursidae* families were found to be susceptible to CAdV-1 infection [3,6,7,8,9,10].

The first case of respiratory disease associated with CAdV-2 infection was reported in 1961 in Canada, when dogs between 8 and 16 months of age who were part of an experimental group for cardiovascular disease research showed dry cough, low-grade fever, loss of appetite, and inflammation and edema of the larynx, pharynx, and trachea [11]. There were no fatalities, and spontaneous resolution occurred. The fact that the viral isolate, nominally Toronto A26/61 (GenBank ID: U77082), was an adenovirus type was inferred from the production of intranuclear inclusion similar to CAdV-1 infection and the sharing of complement-fixing antigens with CAdV-1 [11,12,13]. To date, CAdV-2 infection is widespread in dogs [14,15,16] and included in the multi-etiological syndrome called kennel cough, which mainly affects community dogs [17,18,19]. Antibody cross-reaction between CAdV-1 and CAdV-2 has been exploited in CAdV-2 MLV to protect against CAdV-1 infection [13,20]. Indeed, CAdV-1 infection is currently considered to be effectively controlled in the domestic dog population using CAdV-2 MLV [20,21,22,23,24,25,26], which is successful in protecting against ICH and safer than CAdV-1 MLV [23]. Furthermore, due to cross-reactivity, most of serological tests cannot be used to discriminate the two viral types [25]. Therefore, seroprevalence data reported in the literature as specific to CAdV-1 or CAdV-2 may not provide a true picture of the epidemiological situation and should be reported as CAdV seroprevalence.

CAdV-1 and CAdV-2 belonged to the Adenoviridae family and the *Mastadenovirus* genus [27,28]. Their genome consists of a single linear molecule of double-stranded DNA (dsDNA) of approximately 30.5 and 31.3 kilobase pairs (Kbp) in length for CAdV-1 and CAdV-2, respectively [29]. The CAdV-1 and CAdV-2 genomes displayed around a 75% nucleotide identity [30,31], but they express genomic and biologic differences. The virions are about 80 nm in diameter and are non-enveloped, with capsid of icosahedral symmetry [32,33]. The capsid has 252 capsomers: 240 hexons that cover the surface of the icosahedron and 12 pentons that represent the vertices of the icosahedron. Each penton has a protein structure called fiber that extends outwards and ends with a spherical structure (knob) responsible for cellular tropism [27,31,32]. Differences in the fiber protein structure are responsible for the different tropisms displayed by the two viruses [31]. CAdVs are highly stable and resistant in the environment, and they replicate in the host cell nucleus, producing typical intranuclear inclusion bodies [11,27].

## 2. Canine Adenovirus Type 1 (CAdV-1)

In domestic and wild carnivore hosts belonging to the *Canidae*, *Mustelidae,* and *Ursidae* families, CAdV-1 exhibits tropism for different tissues. Viral pathogenesis was investigated in more detail for domestic dogs than for wild animals; therefore, the available data regarding these aspects refer mainly to the domestic dog. After natural exposure, the virus initially replicates in the tonsils and progressively reaches the regional lymph nodes and the blood, causing viremia. Viremia allows the virus to reach and replicate in different tissues and organs, such as the eyes, liver, kidneys, and other vascular endothelial cells of many tissues, including those of the central nervous system (CNS) [31,34,35,36,37]. In dogs, the liver is recognized as the main target of infection, hence the name infectious canine hepatitis (ICH) for the corresponding disease, characterized by acute necrohemorragic hepatitis [37]. After the acute stage of the disease, corneal edema (“blue eye”), uveitis, and interstitial nephritis may occur as a consequence of circulating immune complex deposition [37]. The incubation period is four to six days after ingestion of infectious material and six to nine days after direct contact with infected dogs [25,38]. Infection in dogs usually occurs with fever, anorexia, conjunctivitis, acute liver failure, jaundice, kidney injury, hemorrhagic enteritis, and in rare cases, neurological signs and “blue eye” [25,31,37,39,40,41,42,43]. In unvaccinated animals or those less than one year old, the clinical signs are more severe, and the mortality rate is high (10–30%), whereas a subclinical course may be observed in vaccinated adults [25,40,42,44]. During the acute stages of the disease, CAdV-1 can be detected from any animal tissues, secretions, and excretions (saliva, urine, feces), while in the absence of chronic hepatic fibrosis, the kidney represents the main site of persistence, with CAdV-1 excreted in the urine for at least 6–9 months post-infection (PI) [37,45]. The histologically striking finding is the presence of large eosinophilic intranuclear inclusion bodies, mainly within the hepatocytes surrounding the necrotic foci but also in epithelial and endothelial cells in other organs [45].

### 2.1. CAdV-1 Infection in Fox Populations

In foxes, CAdV-1 infection is typically associated with injuries to the CNS accompanied by neurological signs, and it is known as “epizootic fox encephalitis” [1,2]. Nevertheless, the majority of foxes and wild animals who laboratory-tested positive for CAdV-1 were asymptomatic [6,46,47], and only some cases of encephalitis, jaundice, depression, and anorexia were reported [2,10,34,36,48].

CAdV-1 infection is frequently reported in red foxes (*Vulpes vulpes*), which appear to be the most susceptible species; consequently, they are the most investigated animals (Table 1). Over the last decades, other fox species infected with CAdV-1, such as the arctic fox (*Vulpes lagopus*), argentic fox (*Urocyon cinereoargenteus*), fennec fox (*Vulpes zerda*), and hoary fox (*Lycalopex vetulus*), have been reported worldwide using direct laboratory assays. Furthermore, potential CAdV-1 infection was detected by serological assays in the gray fox (*Lycalopex griseus*), Indian fox (*Vulpes bengalensis*), island fox (*Urocyon littoralis*), and San Joaquin kit fox (*Vulpes macrotis mutica*), but further studies are needed to distinguish exposure to CAdV-2. The frequency of infection, year of detection, and geographical origin of the fox species testing positive for CAdV-1 are reported, with references, in Table 1.

It has been suggested that like dogs, young red foxes are more susceptible to CAdV-1 infection than adults, and they exhibit more severe clinical signs and higher mortality [34,48]. In fact, Walker et al. [34] reported the fatal cases of four juvenile red foxes who tested positive for CAdV-1 which showed neurological signs and jaundice, while Pacini et al. [63] detected CAdV-1 in 3/12 adult red foxes, without clinical signs or death. Data on clinical manifestations in other fox species are limited, although fatal cases in an adult argentic fox and an adult fennec fox have been reported [53,56]. Several authors reported a higher prevalence of antibodies potentially against CAdV-1 in adult than young foxes belonging to different species [51,52,57,58,59], probably due to the possibility of reinfection during their lifetimes [59]. One study reported a higher seroprevalence in juvenile red foxes in winter (42%) than in autumn (36%), suggesting a correlation to the concomitant breeding season, which allowed more frequent contacts between the animals [67]. However, it should be remembered that the most commonly used serological tests are not able to distinguish antibodies against CAdV-1 and CAdV-2, and that a possible cross-reaction must be taken into account when interpreting these data [51,85].

Histopathological examinations in concomitant CAdV-1 infection revealed lesions in different organs [1,6,34,36,56]. In particular, in the brain, a mild perivascular lymphohistiocytic inflammatory infiltration of the meninges and occasionally, necrosis of the neurons was observed, and in the liver, the random foci of hepatocellular necrosis and fatty change were reported. Furthermore, numerous eosinophilic intranuclear inclusions were reported in the CNS, liver, and kidney. Currently, the few studies reporting brain lesions were carried out on young animals [34,36]. Further studies should be conducted to investigate whether CNS lesions are typical only for young animals, although it is not always possible to carry out a histological examination in wild animals, especially if they are found dead and subjected to tissue autolysis [48,65].

### 2.2. CAdV-1 Infection in Other Wild Animal Species

After its detection in fox populations [2], other animal species, such as coyote, mink, ferret, rabbit, white rat, gray squirrel, guinea pig, domestic cat, sheep, and monkey, were experimentally infected with CAdV-1 [6]. Experimental infections showed that coyotes (*Canis latrans*) had a susceptibility similar to that of foxes, with comparable clinical signs and eosinophilic intranuclear inclusion bodies in the CNS and hepatic cells. Conversely, the other animal species tested were resistant to infection. In subsequent years, natural CAdV-1 infection was reported in mammalian carnivore species belonging to the *Canidae* and *Mustelidae* families, such as wolves (*Canis lupus* and *Chrysocyon brachyurus*), coyotes, raccoons (*Procyon lotor*), badgers (*Meles meles*), Euroasian river otters (*Lutra lutra*), and recently, in minks (*Mustela vison*), through the use of direct or serological assays (Table 1).

CAdV-1 infection in wolves was frequently detected by molecular assays in several geographic areas, such as Brazil [70], Canada [72], France [50], Italy [75,78,81], and Spain [73,82], but clinical signs have only been reported for one wolf positive for CAdV-1, which showed weakness, anorexia, and an intermittent head shaking [50], and in two maned wolves, which showed lethargy, gastrointestinal and neurological signs, corneal opacity, lymphadenomegaly, and persistent bleeding [70]. Although the frequent detection of infection in wolves suggests a susceptibility comparable to that of foxes, on the other hand, the few reports of clinical signs could suggest that CAdV-1 infection occurs subclinically in this species [71,80].

Raccoons seem to be a relatively resistant species, as demonstrated by Green et al. through an experimental inoculation, in which only one infected animal showed typical clinical signs [46], and by the low prevalence of infection detected by molecular (0/48, 0%) and serological (from 0% to 6.9%) assays, as described in the literature [35,90,107]. Furthermore, serological surveys conducted in North America and Japan reported no age-related differences in the susceptibility of raccoons to CAdV infection [89,90,91].

To date, free-ranging coyotes were tested only with serologic assays, detecting prevalence values ranging from 61.8% to 100% in several geographic areas [54,66,84,85,86,87], and in just one study, CAdV-1 was isolated from the liver and kidneys of a coyote [88]. As for foxes, higher seroprevalence values were observed in adults (from 67% to 95%) than in young (from 0% to 44%) coyotes [66,86].

Badgers and otters have been less frequently investigated compared to other wild animal species. Two surveys on badgers were carried out in Italy [63,64], and in only one of these two studies, animals from areas with a high human population density tested positive [63]. In a study conducted in Brazil by de Mello Zanim Michelazzo et al., no otter tested positive [92], whereas in Korea, a Euroasiatic otter that died after prolonged anorexia and weight loss tested positive for CAdV-1 DNA [10]. The low frequency of infection reported for these two species could be due to their low susceptibility to CAdV-1 or the high mortality of the infection, such that infected animals that die in the wild are not found and investigated.

Recently, in a study carried out in China, 30/540 minks tested positive for CAdV-1 with no clinical signs, showing how the virus has evolved to infect species hitherto considered resistant. From the phylogenetic analysis of the fiber gene nucleotide sequence obtained from a CAdV-1 detected in minks, the authors observed a close relationship with viruses identified in Norwegian arctic and red foxes and a distant relationship with viruses infecting Italian dogs, suggesting that CAdV-1 may have been transferred from foxes to minks [93].

In addition to the wild carnivores described above, CAdV-1 infection was also reported in several species belonging to the *Ursidae* family (Table 1). The first indication of a possible infection in a bear dates back to 1950, when a captive polar bear (*Ursus maritimus*) cub from USA with prostrate condition was recovered using a fox encephalitis antiserum intraperitoneally [106]. Subsequently, CAdV-1 was isolated for the first time from two black bear (*Ursus americanus*) cubs with fatal clinical signs [8]. Evidence of widespread circulation of CAdV in bears via the serological method was first reported by Zarnke and Evans [104], who tested 526 grizzly bears (*Ursus arctos horribilis*) from 1973 to 1986 and detected 72/526 (13.7%) positive animals, with no association with the year of sampling. Further serological screening detected CAdV exposition in black bears from the USA [94,95,96,97] and Canada [83], in brown bears (*Ursus arctos*) from Italy [105] and the USA [99], in grizzly bears from the USA [97] and Canada [83], and in polar bears from Canada [83]. Conversely, Vitásková et al. detected no antibodies against CAdV-1 in brown bears from Slovakia [108]. CAdV-1 infection was also detected by viral isolation (VI) or molecular assays in black bears from the USA [88], in brown bears from Spain [98,100] and in grizzly bears from the USA [103].

CAdV-1 infection in bears was reported in association with sialorrhea, ataxia, vomiting, and neurological signs [8,88,103]. The infection appears to cause more severe disease in young animals than in adults; in fact, the detection of CAdV-1 in bear cubs was frequently associated with death [8,98,100,103]. As a result, in surveys carried out on bears using serological assays, adult animals exhibited higher seroprevalence values than did young ones, probably because the former survive the infection, while the latter die, or because adult animals have been repeatedly exposed to the virus [83,94,95,96,97,99,105]. Histological examinations of the dead bears showed necrosis and eosinophilic intranuclear inclusion bodies in the brain, liver, and kidney cells, along with areas of demyelination in the CNS [8,88,100,103].

Serological surveys were also performed in giant pandas (*Ailuropoda melanoleuca*) in China (Table 1), but these animals were subjected to preventive vaccination strategies to minimize the risk of the development of fatal clinical signs; therefore, it was not possible to evaluate the real spread of the infection [101,102].

## 3. Canine Adenovirus Type 2 (CAdV-2)

CAdV-2 replicates in the non-ciliated bronchiolar epithelial cells; in the surface cells of the nasal mucosa, pharynx, and tonsillar crypts; in the mucous cells in the trachea; and in the bronchial and alveolar cells [17,25]. Peak replication is reached by 3–6 days PI; then the viral load declines rapidly (related to antibody production), and CAdV-2 is usually no longer detected after 9 days PI [17,50,109]. In domestic dogs, the virus causes mainly mild respiratory symptoms, such as laryngotracheitis, pharyngitis, and necrotizing bronchitis [11]. When the CAdV-2 infection is associated with secondary bacterial or viral respiratory infections and the animal is not vaccinated or is immunodepressed, it may cause more severe clinical signs, like bronchopneumonia, which may progress to death [15,25,110]. CAdV-2 infection has a high prevalence in canine communities [14,15,16], and the most serious clinical form of infectious tracheobronchitis (ITB), with multiple etiology, is also known as kennel cough [17,37]. CAdV-2 was also isolated in dogs who died after clinical manifestation of pneumonia [110], and rarely, it was detected in feces [19,111] and was sometimes associated with fatal cases of diarrhea [112,113]. The detection of CAdV-2 DNA in intestinal or fecal specimens could be associated with an active digestive tract infection, but ingestion of infected nasal secretions has also been suggested [111,114]. CAdV-2 was also reported in association with neurological signs [115,116]. Histopathologically, red areas of consolidation in the lungs, necrosis of bronchial cells, and eosinophilic intranuclear inclusion bodies are the most frequent findings in course of CAdV-2 infection [25,31,117].

In wild carnivores, potential cases of CAdV-2 infection were reported only in the last twenty years through VI, immunohistochemistry (IHC), or the detection of viral DNA, with variable frequency in red foxes [45,50,62], raccoon dogs [61], wolves [50,75,82], raccoons [50], and in neotropical otters (*Lontra longicaudis*) [92], and in different geographical areas from Europe, Asia, and South America. The frequency of infection, year of detection, and geographical origin of the wild animal species testing positive for CAdV-2 are reported, with references, in Table 1. Furthermore, given the impossibility of the serological assays most commonly used for screening purposes in wildlife populations to distinguish antibodies against CAdV-1 and CAdV-2, respectively, it is possible to hypothesize that other animal species testing seropositive for CAdV were also exposed to CAdV-2 infection (Table 1).

To date, no association with clinical signs has been suggested in either free-living wildlife or zoo animals [50,77,82], except in captive neotropical otters from Brazil, coinfected with canine distemper virus, diagnosed with pneumonia [92]. Histological examinations were performed in the upper respiratory tracts of these otters, but no microscopic alterations were observed, except for signs of immunoreactivity detected by IHC in the peribronchial glands from four animals [92]. Furthermore, CAdV-2 was isolated from two raccoon dogs found dead due to road accidents or diseases and in which no visible clinical signs of CAdV infection, other than hair loss, have been observed [61]. As in domestic dogs, it is possible that the virus circulates mostly asymptomatically in wild carnivores and infects the upper respiratory tract.

## 4. Diagnosis of Infection and Genetic Analysis of CAdV-1 and CAdV-2

Diagnosis of CAdV-1 and CAdV-2 infection can be achieved by using direct methods, which allow for the detection of the virion or its components, and indirect methods, which detect antibodies against viral components produced by the host as a result of infection or exposition. Serological assays are generally useful for understanding the epidemiology of the two CAdV types in wildlife. However, the detection of an antibody titer is indicative of exposure to the pathogen over an indefinite time period and does not indicate an ongoing infection. Furthermore, serological tests are more accessible and rapid than direct assays, but their applicability to wildlife needs to be carefully evaluated, as few tests have been validated for the different wild animal species. The most used indirect methods for the diagnosis of CAdV infection include the virus neutralization (VN) test [48,55,69,74,80,89,94,99], the enzyme-linked immunosorbent assay (ELISA) [58,67], the indirect fluorescence antibody test (IFAT) [15,51,68,108], and the hemagglutination inhibition (HI) assay [13,118,119].

The VN is the serological test most frequently used for screening in wild animals (Table 1), but it is unable to discriminate antibodies against CAdV-1 from those against CAdV-2 [47,57,83,84,85,86,89,94,105], limitations also inherent in the ELISA and IFAT assays [51,58,67]. Since the two CAdV types exhibit different hemagglutination patterns [25], the HI assay is recognized as the only serological test capable of differentiating the presence of antibodies against CAdV-1 compared to CAdV-2 [118]. However, this assay is not used for screening wild animals due to the need for specific erythrocyte cultures for the two viruses [11,120].

The direct diagnostic assays most frequently used for CAdV-1 and CAdV-2 detection are VI on canine kidney cells, such as Madin–Darby canine kidney (MDCK) cells [4,11,15,32,48,50,56,121]; electron microscopy (EM) [15,122]; the hemagglutination test (HA) [118,120]; restriction fragment length polymorphism (RFLP) analysis [113,123,124]; IHC [36,50,125,126]; and molecular assays such as end-point PCR, real-time quantitative PCR (qPCR), and sequencing [15,34,35,41,45,50,53,62,82,93,127,128,129,130,131].

VI assay on MDCK cells was the first diagnostic method adopted for CAdV detection and identification [4,11]. It is an effective method to evaluate the presence, vitality, and pathogenicity of the virus, but it requires long execution times, and it is not alone able to discriminate between CAdV-1 and CAdV-2. In fact, cytopathic effects, like intranuclear inclusion bodies, cells death, and large gaps formed in the cell sheet, were observed in cellular cultures infected by both viruses [11,32,121]. VI can be combined with other methods, such as HA, end-point PCR, qPCR, and sequencing, to discriminate the two viruses [15,48,56,61,81,103,118].

EM found application, especially in past years, for the structural characterization of the viruses identified [10,11,15,122] but without the possibility of differentiating the two viral types. Therefore, it was combined with other diagnostic tests such as molecular methods or serology with monoclonal antibodies [10,15].

HA and RFLP assays may be used to differentiate the two CAdV types. HA exploits the ability of CAdV to adsorb the erythrocytes of different animals, such as rats, guinea pigs, fowls, pigs, and mice [15]. Furthermore, the different hemagglutination patterns shown by CAdV-1 and CAdV-2 allow for the HA assay to differentiate the two viruses [118]. Specifically, CAdV-1 agglutinates the erythrocytes of humans (O-type), albino rats, guinea pigs, and chickens [120], whereas CAdV-2 can agglutinate the erythrocytes of chickens, humans (O-type), and albino rats, but cannot agglutinate the erythrocytes of mice, guinea pigs, and fowls [11,15,31]. RFLP assay uses specific restriction enzymes that catalyze DNA strand cutting in correspondence to specific nucleotide sequences. The two CAdV types were discriminated using specific restriction endonucleases, such as *BglI*, *EcoRI*, and *HincII* [113,123,124]. To date, this test is little used due to its low sensitivity and long execution times.

IHC assay allows for the histological detection of the presence of CAdV antigens in a tissue sample using enzyme-linked antibodies. The antigen–antibody binding activates the enzyme in the tissue sample, which can be seen under a microscope. The two CAdV types should be differentiated by the use of the monoclonal anti-adenovirus antibody but not by the use of polyclonal antibodies [36,92,100,103,125,126]. It is a useful method for confirming the tissue infection, but it is not a sensitive technique. In fact, a discrepancy between IHC and end-point PCR results was reported for the CAdV-1 detection in kidney samples [65].

In recent years, end-point PCR, qPCR, and sequencing have been used more extensively due to their high sensitivity [65,132], low costs, short time of execution, and their ability to discriminate the two viral types [127,128,129,130] and to genetically analyze the identified virus [41,43,50]. An end-point PCR assay capable of differentiating CAdV-1 and CAdV-2 by amplifying a fragment of the E3 gene and the flanking genes of different lengths for the two viruses (508 bp for CAdV-1 and 1030 bp for CAdV-2) was developed by Hu et al. and validated for different biological samples [127,128]. This assay was widely used for diagnostic and screening purposes [49,61,63,65,76,81,133]. In recent years, qPCR assays were also developed to detect, quantify, and differentiate CAdV-1 and CAdV-2 DNA using SYBR Green chemistry, with melting temperature analysis [129] or virus-specific probes [130].

Liver, spleen, lymph node, kidney, brain, intestine, urine, and feces are the most used biological samples for the detection of CAdV-1 DNA [36,45,56,62,63,64,65,72,75,93,100,103,134]. In contrast, CAdV-2 DNA in domestic dogs was mainly detected in ocular swabs, lung sections, respiratory secretions, and in fecal samples [14,25,92]. In wild animals, CAdV-2 DNA was occasionally detected in fecal samples [45,79], but it is not known whether this resulted as a consequence of replication in the intestinal tract or from the ingestion of respiratory secretions. Furthermore, CAdV-2 DNA was also detected in the spleen, liver, and intestines [50,61,62,77,115], but this aspect requires further investigation. CAdV-1 and CAdV-2 DNA were also detected in tongue samples from wolves [75,76,78] but with concomitant negative results via IHC assay [78]. Although the use of this biological matrix could find applications in the detection of enteric viruses from carcasses of animals subjected to autolysis [135], further studies are needed to evaluate whether CAdV replicates in the lingual tissue or is only present in the saliva.

In several studies conducted on wildlife, the PCR products obtained from the amplification of the E3 gene and the flanking genes were sequenced and analyzed [35,45,49,56,63,76,79,100,134]. However, to genetically characterize or phylogenetically analyze the virus detected, other end-point PCR assays targeting different genes codifying for structural or non-structural proteins were used, i.e., hexon gene [49,50,61,62,64,65,72,75,78,103,134], fiber gene [49,61,62,72,75,78,93,134], penton base gene [61], polymerase gene [34,36], E4 gene [65], E1B 19K/small T antigen gene [53], 100K protein gene [93], and ORF30 gene [134]. The nucleotide sequences of the CAdV genes currently available in the GenBank database (https://www.ncbi.nlm.nih.gov/nucleotide/, accessed on 20 August 2024), together with a few entire viral genome sequences, are reported in Table 2. In particular, the complete genome of two CAdV-1 identified in wolves [50,81] and one CAdV-2 identified in a raccoon dog [61] were obtained by next-generation sequencing (NGS).

In general, despite the small number of CAdV-2 nucleotide sequences available compared to the number for CAdV-1, sequence analysis allows to easily distinguish CAdV-1 from CAdV-2, but few nucleotide mutations differentiating strains belonging to the CAdV-1 viral type have been reported. The E3 gene and flanking regions were frequently sequenced and analyzed for convenience, as they are the product obtained from the most widely used diagnostic end-point PCR [127], but few sporadic nucleotide mutations were reported in this genetic fragment [35,45]. An exception was the identification of an additional 22-nucleotide tract in the noncoding region between the E3 gene and U-exon gene that differentiate the CAdV-1 detected in some red and arctic foxes from Norway and Svalbard, respectively [49]. In contrast, in the deduced hexon and fiber proteins, some amino acid residues showed variability between the CAdV-1 identified in different wild animal species and geographical areas. Different amino acid residues were reported in codons 99, 138, 234, and 388 of the hexon-deduced protein and codons 23, 110, 284, 305 and 319 of the fiber-deduced protein [49,50,62,64,72,75,78,81]. The phylogeny generally showed a slight clusterization of the CAdV-1 to date identified in wildlife and domestic dogs on the basis of the geographical origin rather than on the host species [36,45,49,62,64,65,72,79,100], suggesting that the same viruses infect both the wild animals and the domestic dogs, supporting the hypothesis of a wild to domestic (or vice versa) transmission.

In particular, phylogenetic trees constructed from the concatenated sequences of the hexon and fiber genes showed a clustering of the CAdV-1 recently identified in European wildlife into two groups. One group was composed by viral strains identified in foxes from Norway and Svalbard, characterized by the distinctive residue 388-aspartate (Asp) in the deduced hexon protein [49]. The second group was composed of viral strains identified in domestic and wild canids from Italy and France, characterized by the distinctive residue 388-serine (Ser) in the deduced hexon protein [62,75,78]. These two clusters were distinguishable from all other available sequences belonging to older CAdV-1 identified worldwide in domestic dogs and to CAdV-1 recently detected in gray wolves from Northern Canada, characterized by the distinctive residue 388-asparagine (Asn) in the deduced hexon protein [72]. These results suggested that the amino acid residue in position 388 of the hexon protein could be able to differentiate CAdV-1 belonging to some different geographical regions (Table 3) [41,49]. Balboni et al. [49] also reported that a change in amino acid position 388 of the hexon protein could determine a change in predicted immunogenicity. Other nucleotide or amino acid substitutions unique to the wild animal populations investigated may indicate that CAdV-1 has been circulating for a long time in different and segregated geographical areas [49,72]. Furthermore, phylogenetic analysis based on the fiber gene located the first CAdV-1 sequenced from minks in a separate branch closely related to the previously mentioned viral strains identified in foxes from Norway and Svalbard [93]. Hou et al. [93] also analyzed the 100K protein gene, encoding one of the most abundant non-structural proteins in adenovirus infection, evidencing a strict relationship between the CAdV-1 of foxes and dogs and a separate grouping for mink CAdV-1.

To date, rare CAdV-2 sequences generated from wild animals were included in phylogenetic analyses. Recently, two CAdV-2 were sequenced from wild raccoon dogs in Korea, showing a close relationship with the traditional Toronto A26/61 strain and suggesting extreme stability of this virus over time in different hosts and over large geographical distances [61].

The evolution of adenoviruses shows a long-term cospeciation with the hosts, characterized by usually asymptomatic or mildly asymptomatic infections, associated with occasional switches between hosts that can determine more severe clinical manifestations [136]. The phylogeny of the *Mastadenovirus* genera evidenced strict relationships between CAdV, skunk adenovirus (SkAdV), and bat adenoviruses (BtAdVs) [136]. Genetically different adenoviruses were identified in a wide range of bat species [137], supporting a long coevolution of these viruses with their hosts. In particular, CAdV-1 and CAdV-2, as well as SkAdV, showed a close phylogenetic relationship to BtAdVs, supporting the origin of these viruses from a common ancestor [138,139], or that CAdV may have originated by interspecies transfer of a BtAdV [138,140]. Kohl et al. [138] suggested that the relatively recent adaptation of these viruses to new carnivorous mammalian hosts may be consistent with the higher pathogenicity of CAdV compared to that of other mastadenoviruses and the ease with which they can cross the host species barrier.

## 5. The Interface Between Wildlife and Domestic Animals

To date, the wildlife–domestic animals’ interface is a topic of great interest, especially in the study of the epidemiology of infectious diseases from a One Health perspective. In fact, wildlife can play a crucial role in the maintenance of infectious agents in the wild and peri-urban environments and can transmit them to domestic animals [45,58]. At the same time, domestic animals can represent a source of infection for threatened wild animal species and cause a further reduction in population density [105]. CAdV infection could affect wildlife conservation, influencing population dynamics and species survival. From this perspective, Clifford et al. [59] reported high rates of CAdV seroprevalence in island foxes in the California Channel Islands, whose survival is close to threatened. Although it has not been proven that CAdV infection was directly responsible for the deaths of the island foxes, the authors suggest that it may have contributed to the worsening health conditions of the animals, such as pups, which often die in dens, their deaths remaining uninvestigated. The epidemiology of CAdV-1 and CAdV-2 evidenced how the two animal populations are closely connected to each other. CAdV-1 is shed in feces, saliva, urine, and respiratory secretions for a variable length of time [31], extending from six to nine months PI for urinary shedding [25], while CAdV-2, exhibiting a predominantly respiratory tropism, is shed in nasal secretions and droplets [17,25] and potentially, in feces [111,114]. Animals can be infected by direct contact with symptomatic or asymptomatic animals or by indirect contact with infected secretions and excretions or contaminated fomites in the environment [25,37]. The possibility for the transmission of CAdV between wildlife and domestic animals through direct contact with infected animals is potentially minimal, considering that the two populations display different lifestyles and habits. Nevertheless, it should be underlined that several anthropogenic drivers, such as agricultural intensification measures, farms, deforestation, and the fragmentation of ecosystems and wildlife habitats encourage contacts at the domestic–wildlife ecosystem interface. The increased movement of people, animals, food, and trade associated with accelerated urbanization often provides favorable grounds for the emergence of infectious diseases, including zoonoses. Progressive urbanization, especially when unplanned and with poor infrastructure, creates novel and diverse contacts among wildlife and domestic animal species, although, due to the high stability of the two CAdV types, which can persist for a long time in the environment [27,32], the main route of transmission between wildlife and domestic populations is probably indirect [141]. The transmission from wild to domestic (or vice versa) animals is sustained by the phylogeny of the CAdV-1 sequenced to date, which revealed a close relationship between viruses identified in wild animals and domestic dogs, as well by the identical amino acid profiles of the viruses detected in the two animal populations [36,45,49,62,64,65,72,79,100]. Furthermore, viral transmission to wild animals via domestic dogs is supported by the high seroprevalence detected in wild animals living in the urban and peri-urban areas [74]. Consequently, both animal populations can mutually influence the epidemiology of CAdV, and for this reason, they must be considered as a whole, in a diverse but communicating environment. Indeed, CAdV circulates autonomously in wild animal species but can also be introduced in them through contact with domestic dogs. At the same time, CAdV continues to circulate autonomously in domestic dogs, despite the virtual disappearance of ICH from regions where vaccination has been performed for many years because the vaccine is not completely protective. Furthermore, recurrent outbreaks, particularly those reported in animal shelters and breeding kennels, are associate with the illegal trade of dogs from Eastern Europe, which are often not adequately vaccinated [142]. A greater awareness of the current impact on the transmission of infectious agents caused by frequenting wild environments and contact with wild animals, combined with the development of advanced vaccines that could be used in domestic and endangered wild animals, would reduce the threat to wildlife, preserve biodiversity, and reduce clinical cases in domestic animals.

## 6. Conclusions

CAdV-1, discovered almost a century ago, can be considered an ancient and well-known virus. Due to the widespread use of modified live vaccines to control canine adenoviral infections and subsequently reduce disease incidence, CAdV-1 is often neglected by clinicians, and its pathogenic role and the clinical form related to CAdV-1 infection in wild animal species are unknown. The proof of this is the limited number of studies available in the literature and CAdV-1 genomic sequences detected in wild animal species available in open databases. Nonetheless, the available data show that CAdV-1 circulates widely in various wild animal species and that transmission of the virus in the interface between wildlife and domestic animals is a frequent event. Therefore, large free-roaming populations of wild animals can act as mixing vessels for CAdV-1, and long-time cospeciation with hosts predisposes the development of mostly asymptomatic infections, resulting in transmission of the virus from wildlife to dogs. Furthermore, although wild animals are usually subject to asymptomatic infections, cases of serious and fatal diseases have been documented, with possible effects on the conservation of the species. In constrast, CAdV-2 infection is well known in domestic dogs but reported only recently and sporadically in some wild carnivores, with little data regarding its pathogenic role in these animal species. The circulation of CAdV-2 in the wildlife limits the reliability of CAdV-1 seroprevalence assessment, as serological tests are mostly unable to distinguish antibodies against CAdV-1 and CAdV-2, respectively.

**Figure 1:**
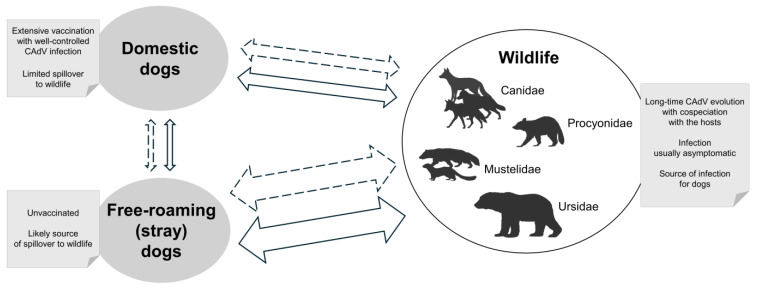
Epidemiology and transmission of CAdV infection at the interface between wildlife and domestic animals. Continuous arrows: direct transmission; dotted arrows: indirect transmission.

The interest related to the circulation of CAdV, in particular CAdV-1, in wildlife is mainly due to the impact that this virus could have on the conservation of animal populations, with potentially drastic numerical reductions. Although CAdV-1 is considered a relatively stable virus, both genetically and antigenically, its spread among wild animals may lead to different scenarios. The most frequent scenario that can occur is a constant endemic circulation of the virus in geographical areas and in wild animal species historically subject to infection. In this case, animals will be largely asymptomatic but still able to shedding significant amounts of the virus in the environment. However, sporadic outbreaks and rare cases of serious or lethal illness are possible, probably as a consequence of a marked individual sensitivity or the involvement of immunocompromised subjects. In this regard, young wild animals would be at a high risk of developing severe clinical forms due to the acquisition of incomplete immunity. In the interface with wild animals, dogs may show the highest risk of developing clinical disease. In fact, under conditions of high viral circulation, dogs that frequent the wild environment, such as hunting and truffle dogs, dogs living in rural environments, or dogs that follow their owners in recreational activities outside the urban environment, can be infected and develop severe clinical signs, especially if not properly vaccinated. In particular, stray dogs are at greater risk of contracting the infection, as they are not vaccinated and may frequently approach wild environments. Conversely, a rapid spread of CAdV-1, with diffuse mortality and serious effects on wild animal conservation, particularly in at-risk species, may occur when the virus first infects species or segregated populations that have never been exposed to it. This scenario may be a consequence of two events that determine a lack of immune protection in the affected animals. First, different viral variants could emerge. Indeed, although the CAdV genome is generally stable, reassortment events or the accumulation of mutations leading to antigenic variations cannot be excluded. New viral variants could spread and cause disease in naive animal populations lacking adequate immune protection. In the second case, already circulating viruses could reach animal populations never previously exposed to the infection because they are geographically isolated. In these cases, domestic dogs that frequent the wild environment can represent an important route of infection and viral introduction. In this regard, in developed countries, most CAdV infections in domestic dog populations are well controlled by extensive vaccination; therefore, the scenario of diseases from domestic dogs spilling over to wildlife may be limited. Of greater concern are developing or underdeveloped areas, where the mutual spilling from unvaccinated free-roaming (stray) dogs to wildlife may be more frequent. Figure 1 shows a graphical representation of the epidemiology and transmission of CAdV infection at the interface between wild and domestic animals.

This review, reporting all the data currently available on CAdV-1 and CAdV-2 infection in wild animals, underlines the importance of these pathogens in the conservation of wildlife and of these hosts in the transmission of viruses to the domestic dog. To date, the data available are still fragmentary. In particular, available studies in the literature regarding CAdV-1 and CAdV-2 infection in wild animal species are sporadic for several hosts, are conducted in different geographical areas and times, use different diagnostic methods, and the genetic analysis of the identified viruses is insufficient. Consequently, the data available do not allow for the contextualization of prevalence trends in different geographical areas or wild animal species, nor do they provide adequate information on the viral transmission or coevolution of the two viruses in the host species. Further studies are desirable to fill the knowledge gap that affects these two viral pathogens.

## Figures and Tables

**Table 1:** Prevalence of canine adenovirus type 1 and 2 infection in wild animal species reported in the literature.

Animal Species	Geographical Origin	Year *	Diagnostic Methods	Samples Tested	Virus and Prevalence Detected	References
FOXES						
Arctic fox (*Vulpes lagopus*)	Norway	1997–2002	PCR	Liver, kidney, lymph node, and spleen	CAdV-1: 7/10 (70%)	[49]
	France	2006–2016	VN	Serum	CAdV: 3/7 (42.8%)	[50]
	Norway	1995–2003	IFAT	Serum	CAdV: 41%	[51]
	Norway	1999–2001	VN	Serum	CAdV: 14/37 (37.8%)	[52]
Argentic fox (*Urocyon cinereoargenteus*)	USA	2005	PCR + histology	Liver	CAdV-1: 1	[53]
	USA	1992–1995	VN	Serum	CAdV: 88%	[47]
	USA	1989	VN	Serum	CAdV: 10/18 (55.6%)	[54]
	USA	1978–1979	VN	Serum	CAdV: 3/32 (9%)	[55]
Fennec fox (*Vulpes zerda*)	Egypt	2013	PCR + VI	Liver, kidney, lymph node, spleen, lung, brain, heart, and intestine	CAdV-1: 1	[56]
Gray fox (*Lycalopex griseus*)	Argentina	1998–2001	VN	Serum	CAdV: 4/56 (7.1%)	[57]
Hoary fox (*Lycalopex vetulus*)	Brazil	2023	IHC + PCR	Liver, brain, and eye	CAdV-1: 1	[36]
Indian fox (*Vulpes bengalensis*)	India	2006–2007	ELISA	Serum	CAdV: Males 62% and Females 40%	[58]
Island fox (*Urocyon littoralis*)	USA	2001–2002	VN	Serum	CAdV: 118/309 (38.2%)	[59]
	USA	1988	VN	Serum	CAdV: 97%	[60]
Raccoon dog (*Nyctereutes procyonoides*)	Korea	2017–2020	PCR + full-length genome sequencing + VI	Liver, kidney, lung, and intestine	CAdV-2: 2/105 (1.9%)	[61]
Red fox (*Vulpes vulpes*)	Italy	2022–2023	qPCR + PCR	Spleen, intestine, and kidney	CAdV-1: 2/126 (1.59%) CAdV-2: 1/126 (0.79%)	[62]
	Italy	2020–2021	PCR	Feces	CAdV-1: 3/12 (25%)	[63]
	Italy	2014–2020	PCR	Spleen and intestine	CAdV-1: 4/232 (1.7%)	[64]
	Norway	2007–2009	PCR	Liver and spleen	CAdV-1: 7/10 (70%)	[49]
	Italy, UK, Germany	2017	PCR	Kidney	CAdV-1: Italy 10/36 (28%), UK 8/21 (38%), and Germany 1/29 (3%)	[65]
	France	2015	VN + qPCR	Liver, kidney, spleen, bladder, and urine	CAdV-2: 2	[50]
	Norway	2004–2009	IFAT	Serum	CAdV: 46%	[51]
	Germany	2015–2016	PCR	Liver, lung, and brain	CAdV-1: 11/97 (11%)	[35]
	UK	2011–2013	PCR	Liver, lymph node, spleen, lung, brain, and heart	CAdV-1: 4	[34]
	Italy	2011	PCR	Feces	CAdV-1: 2/32 (6.3%) CAdV-2: 1/32 (3%)	[45]
	Norway	1994–1995 2002–2005	VN	Serum	CAdV: 31/52 (59.6%)	[52]
	UK	1995–200	VN + VI	Serum and liver	CAdV-1: 3	[48]
	USA	1993–2005	VN	Serum	CAdV: 4/9 (44.4%)	[66]
	Australia	1980–1984 1990–1994 1991–1994	ELISA	Serum	CAdV: 308/1326 (23.2%)	[67]
	Germany	1991–1995	IFAT + VN	Serum	CAdV: 17/485 (3.5%)	[68]
	USA	1989	VN	Serum	CAdV: 47/47 (100%)	[54]
	USA	1978–1979	VN	Serum	CAdV: 2/57 (3%)	[55]
San Joaquin kit fox (*Vulpes macrotis mutica*)	USA	1981–1982 and 1984	VN	Serum	CAdV: 1981–1982 1/16 (6%) and 1984 6/29 (21%)	[69]
WOLVES						
Maned wolf (*Chrysocyon brachyurus*)	Brazil	2021	qPCR	Blood	CAdV-1: 2	[70]
	Brazil	2003–2008	VN	Serum	CAdV: 13/14 (92.9%)	[71]
Timber wolf (*Canis lupus occidentalis*)	France	2015	qPCR	Urine	CAdV-2: 1	[50]
Wolf (*Canis lupus*)	Canada	2022	PCR	Spleen	CAdV-1: 3/303 (1%)	[72]
	Spain	2009–2018	PCR	Spleen	CAdV-1: 21/149 (14%)	[73]
	North America	2021	VN	Serum	CAdV: 58/63 (92.1%)	[74]
	Italy	2017–2019	qPCR+ PCR	Spleen and tongue	CAdV-1: 2/23 (8.7%) CAdV-2: 2/23 (8.7%)	[75]
	Italy	2017–2020	qPCR + PCR	Spleen and tongue	CAdV-2: 3/56 (5.4%)	[76]
	Italy	2016	qPCR	Liver, spleen, and lung	CAdV-2: 1	[77]
	Italy	2014	qPCR + PCR	Liver and tongue	CAdV-1: 1	[78]
	Italy	2017	PCR	Feces	CAdV-2: 3/20 (15%)	[79]
	France	2009–2015	VN + qPCR + full-length genome sequencing + VI	Serum, liver, spleen, and intestine	CAdV-1: 1 CAdV: 12/19 (63.2%)	[50]
	USA	2007–2013	VN	Serum	CAdV: Adults 88% and Pups 45%	[80]
	Italy	2015	PCR + VI	Liver	CAdV-1: 1	[81]
	Spain	2010–2013	VN + PCR	Serum and spleen	CAdV-1: 70% CAdV-2: 5%	[82]
	Norway	1998–2007	VN	Serum	CAdV: 63/93 (67.7%)	[52]
	USA	1997–2007	VN	Serum	CAdV: 195/209 (93.3%)	[66]
	Canada	1984–2001	VN	Serum	CAdV: 89%	[83]
COYOTES						
Coyote (*Canis latrans*)	USA	2011	VN	Serum	CAdV: 20/28 (71%)	[84]
	USA	1997–2007	VN	Serum	CAdV: 63/102 (61.8%)	[66]
	USA	1997–2001	VN	Serum	CAdV: 94/122 (77%)	[85]
	USA	1997–1999	VN	Serum	CAdV: 43/67 (64.2%)	[86]
	USA	1985–1990	VN	Serum	CAdV: 68%	[87]
	USA	1989	VN	Serum	CAdV: 13/13 (100%)	[54]
	USA	1983	IHC + VI	Liver and kidney	CAdV:1	[88]
RACCOONS						
Raccoon (*Procyon lotor*)	France	2015	qPCR	Urine	CAdV-2: 1	[50]
	Japan	2004–2006 2009–2010	VN	Serum	CAdV: 2–6%	[89]
	USA	2002–2007	VN	Serum	CAdV: 6.9%	[90]
	USA	1973	VN	Serum	CAdV: 6/50 (12%)	[91]
	USA	1943	Viral inoculation via intraocular route		CAdV-1: 1	[46]
BADGERS						
Badger (*Meles meles*)	Italy	2020–2021	PCR	Feces	CAdV-1: 2/6 (33.3%)	[63]
MARTENS						
Fisher (*Martes pennanti*)	Canada	1984–2001	VN	Serum	CAdV: 4%	[83]
OTTERS						
Eurasian river otter (*Lutra lutra*)	Korea	2007	PCR	Liver	CAdV-1: 1	[10]
Neotropical otter (*Lontra longicaudis*)	Brazil	2022	IHC	Peribronchial glands	CAdV-2: 4/6 (66.6%)	[92]
MINKS						
Mink (*Mustela lutreola*)	China	2023	PCR	Anal swabs	CAdV-1: 30/540 (5.5%)	[93]
BEARS						
Black bear (*Ursus americanus*)	USA	2014–2016	VN	Serum	CAdV: 6/87 (6.9%)	[94]
	USA	1999–2011	VN	Serum	CAdV: 7/82 (8.5%)	[95]
	Canada	1984–2001	VN	Serum	CAdV: 8%	[83]
	USA	1993–1995	VN	Serum	CAdV: 4/66 (6%)	[96]
	USA	1988–1991	VN	Serum	CAdV: 3/40 (7.5%)	[97]
	USA	1983	IHC + VI	Liver and kidney	CAdV-1: 3	[88]
	USA	1979	VN + VI	Serum and brain	CAdV-1: 2	[8]
Brown bear (*Ursus arctos*)	Spain	1998–2023	qPCR	Liver and brain	CAdV-1: 3/53 (5.6%)	[98]
	USA	2013–2016	VN	Serum	CAdV: 28/155 (18%)	[99]
Eurasian brown bear (*Ursus arctos arctos*)	Spain	1998–2018	qPCR + IHC	Liver	CAdV-1: 4/21 (19%)	[100]
Giant panda (*Ailuropoda melanoleuca*)	China	1994–2005	VN	Serum	CAdV: 26/92 (20 vaccinated)	[101]
	China	1992	VN	Serum	CAdV: 4/8 (50%)	[102]
Grizzly bear (*Ursus arctos horribilis*)	USA	2015	PCR + histology + IHC	Brain	CAdV-1: 1	[103]
	USA	1988–1991	VN	Serum	CAdV: 68/480 (14.2%)	[97]
	USA	1973–1986	VN	Serum	CAdV: 72/526 (13.7%)	[104]
Marsican brown bear (*Ursus arctos marsicanus*)	Italy	2004–2009	VN	Serum	CAdV: 2/22 (10%)	[105]
Polar bear (*Ursus maritimus*)	Canada	1984–2001	VN	Serum	CAdV: 17%	[83]
	North America	1950	Administration of anti-CAdV-1 serum		CAdV-1: 1	[106]

For the serological assays that cannot distinguish the two viral types, only CAdV seroprevalence was reported, without specifying the viral type, even if the article cited reported a distinction. ELISA: enzyme-linked immunosorbent assay. IHC: immunohistochemistry. IFAT: indirect immunofluorescence antibody test. PCR: polymerase chain reaction. qPCR: real-time polymerase chain reaction. UK: United Kingdom. USA: United States of America. VI: viral isolation. VN: virus neutralization test. * Year of sampling or of publication.

**Table 2:** Canine adenovirus type 1 and 2 sequenced and reported in wild animals, available in the GenBank database (https://www.ncbi.nlm.nih.gov/nucleotide/, accessed on 20 August 2024).

Virus	Strain	Geographic Origin	Host Species	Organs	Viral Gene Sequenced	GenBank ID	Reference
CAdV-1	15195	UK	Red fox (*Vulpes vulpes*)	Liver	Hexon, fiber, E3, ORF30	KU755709, KU755760, KU755723, KU755739	[134]
CAdV-1	15346	UK	Red fox (*Vulpes vulpes*)	Kidney	Hexon, fiber, E3, ORF30	KU755708, KU755755, KU755727, KU755744	[134]
CAdV-1	15620	UK	Red fox (*Vulpes vulpes*)	Kidney	Hexon, fiber, E3, ORF30	KU755703, KU755761, KU755725, KU755741	[134]
CAdV-1	15622	UK	Red fox (*Vulpes vulpes*)	Kidney	E3	KU755728	[134]
CAdV-1	15705	UK	Red fox (*Vulpes vulpes*)	Kidney	Fiber	KU755759	[134]
CAdV-1	16036	UK	Red fox (*Vulpes vulpes*)	Liver	Hexon, E3, ORF30	KU755707, KU755726, KU755740	[134]
CAdV-1	16432	UK	Red fox (*Vulpes vulpes*)	Kidney	Hexon	KU755705	[134]
CAdV-1	16606	UK	Red fox (*Vulpes vulpes*)	Liver	E3, ORF30	KU755729, KU755738	[134]
CAdV-1	17066	UK	Red fox (*Vulpes vulpes*)	Liver	Hexon, fiber, E3, ORF30	KU755702, KU755756, KU755724, KU755745	[134]
CAdV-1	17154	UK	Red fox (*Vulpes vulpes*)	Liver	Hexon, fiber, E3, ORF30	KU755706, KU755757, KU755722, KU755742	[134]
CAdV-1	17157	UK	Red fox (*Vulpes vulpes*)	Liver	Hexon, fiber, E3, ORF30	KU755704, KU755758, KU755721, KU755743	[134]
CAdV-1	010515/5	UK	Red fox (*Vulpes vulpes*)	Kidney	E3	KU755720	[134]
CAdV-1	020215/1	UK	Red fox (*Vulpes vulpes*)	Spleen	ORF30, fiber	KU755737, KU755753	[134]
CAdV-1	030415/1	UK	Red fox (*Vulpes vulpes*)	Liver	Hexon, fiber, E3	KU755701, KU755749, KU755719	[134]
CAdV-1	061014/2	UK	Red fox (*Vulpes vulpes*)	Liver	Hexon, fiber, E3, ORF30	KU755700, KU755754, KU755718, KU755735	[134]
CAdV-1	090315/1	UK	Red fox (*Vulpes vulpes*)	Liver	E3, fiber	KU755717, KU755751	[134]
CAdV-1	090315/2	UK	Red fox (*Vulpes vulpes*)	Liver	Hexon, fiber, E3, ORF30	KU755699, KU755748, KU755716, KU755736	[134]
CAdV-1	09-13F	Italy	Red fox (*Vulpes vulpes*)	Feces	E3	JX416838	[45]
CAdV-1	111114/1	UK	Red fox (*Vulpes vulpes*)	Liver	Hexon, fiber, E3, ORF30	KU755698, KU755752, KU755715, KU755734	[134]
CAdV-1	113-5K	Italy	Red fox (*Vulpes vulpes*)	Kidney	E3	JX416840	[45]
CAdV-1	113-5L	Italy	Red fox (*Vulpes vulpes*)	Liver	E3	JX416839	[45]
CAdV-1	201114/1	UK	Red fox (*Vulpes vulpes*)	Liver	Hexon, fiber, E3, ORF30	KU755697, KU755746, KU755714, KU755733	[134]
CAdV-1	201114/2	UK	Red fox (*Vulpes vulpes*)	Liver	Hexon, E3, ORF30	KU755696, KU755713, KU755732	[134]
CAdV-1	201115/2	UK	Red fox (*Vulpes vulpes*)	Liver	Fiber	KU755747	[134]
CAdV-1	220515/1	UK	Red fox (*Vulpes vulpes*)	Liver	Hexon, E3, ORF30	KU755695, KU755712, KU755731	[134]
CAdV-1	300115/2	UK	Red fox (*Vulpes vulpes*)	Liver	Hexon, fiber, E3, ORF30	KU755694, KU755750, KU755711, KU755730	[134]
CAdV-1	300115/3	UK	Red fox (*Vulpes vulpes*)	Kidney	Hexon, E3	KU755693, KU755710	[134]
CAdV-1	452/2017	Italy	Wolf (*Canis lupus*)	Spleen	Hexon, fiber	MW829199, MW829200	[75]
CAdV-1	51.20-118	Italy	Red fox (*Vulpes vulpes*)	Spleen	Hexon	OL323110	[64]
CAdV-1	51.20-213	Italy	Red fox (*Vulpes vulpes*)	Spleen	Hexon	OL323113	[64]
CAdV-1	51.20-28	Italy	Red fox (*Vulpes vulpes*)	Spleen	Hexon	OL323111	[64]
CAdV-1	51.20-93	Italy	Red fox (*Vulpes vulpes*)	Spleen	Hexon	OL323112	[64]
CAdV-1	602-01-2007-spleen	Norway	Red fox (*Vulpes vulpes*)	Spleen	E3	MF344652	[49]
CAdV-1	602-02-2008-spleen	Norway	Red fox (*Vulpes vulpes*)	Spleen	E3	MF344653	[49]
CAdV-1	602-03-2008-spleen	Norway	Red fox (*Vulpes vulpes*)	Spleen	E3	MF344654	[49]
CAdV-1	602-04-2008-spleen	Norway	Red fox (*Vulpes vulpes*)	Spleen	E3	MF344655	[49]
CAdV-1	602-05-2008-spleen	Norway	Red fox (*Vulpes vulpes*)	Spleen	E3	MF344656	[49]
CAdV-1	602-06-2008-spleen	Norway	Red fox (*Vulpes vulpes*)	Spleen	E3	MF344657	[49]
CAdV-1	602-07-2008-spleen	Norway	Red fox (*Vulpes vulpes*)	Spleen	E3, hexon, fiber	MF344658, MF344666, MF344672	[49]
CAdV-1	603-05-2001/02-lymph node	Norway	Arctic fox (*Vulpes lagopus*)	Lymph node	E3	MF344659	[49]
CAdV-1	603-06-1997/98-liver	Norway	Arctic fox (*Vulpes lagopus*)	Liver	E3, hexon, fiber	MF344660, MF344667, MF344673	[49]
CAdV-1	603-07-1997/98-kidney	Norway	Arctic fox (*Vulpes lagopus*)	Kidney	E3, hexon, fiber	MF344661, MF344668, MF344674	[49]
CAdV-1	603-10-1999/00-spleen	Norway	Arctic fox (*Vulpes lagopus*)	Spleen	E3, hexon, fiber	MF344662, MF344669, MF344675	[49]
CAdV-1	603-11-2001/02-kidney	Norway	Arctic fox (*Vulpes lagopus*)	Kidney	E3	MF344663	[49]
CAdV-1	603-12-2001/02-spleen	Norway	Arctic fox (*Vulpes lagopus*)	Spleen	E3, hexon, fiber	MF344664, MF344670, MF344676	[49]
CAdV-1	603-13-1999/00-lymph node	Norway	Arctic fox (*Vulpes lagopus*)	Lymph node	E3, hexon, fiber	MF344665, MF344671, MF344677	[49]
CAdV-1	874-2014-tongue	Italy	Wolf (*Canis lupus*)	Tongue	Hexon, fiber	MH105809, MH105810	[78]
CAdV-1	CAdV/badger/153/IT	Italy	Badger (*Meles meles*)	Feces	E3	OP851366	[63]
CAdV-1	CAdV/fox/894/IT	Italy	Red fox (*Vulpes vulpes*)	Feces	E3	OP851367	[63]
CAdV-1	CAdV-1 1798/2022	Italy	Red fox (*Vulpes vulpes*)	Spleen	Hexon, fiber	PP551652, PP551653	[62]
CAdV-1	CAdV-1 ITL2015	Italy	Wolf (*Canis lupus*)	Liver	Complete genome	KX545420	[81]
CAdV-1	CAV IAL FOX	Brazil	Hoary fox (*Lycalopex vetulus*)	Liver	Polymerase	ON667908	[36]
CAdV-1	CC 24-A-05	USA	Gray fox (*Urocyon cinereoargenteus*)	Liver	E1B 19K	EF611185	[53]
CAdV-1	NT14-200	Spain	Brown bear (*Ursus arctos arctos*)	Liver	E3	MH469715	[100]
CAdV-1	NWT-W110	Canada	Wolf (*Canis lupus*)	Spleen	Hexon, fiber	OK546122, OK546125	[72]
CAdV-1	NWT-W167	Canada	Wolf (*Canis lupus*)	Spleen	Hexon, fiber	OK546123, OK546126	[72]
CAdV-1	NWT-W85	Canada	Wolf (*Canis lupus*)	Spleen	Hexon, fiber	OK546121, OK546124	[72]
CAdV-1	RZ-4/2023	China	Mink	Feces	100 K protein	OQ981364	[93]
CAdV-1	wolf/835/2015/FRA	France	Wolf (*Canis lupus*)	Liver, spleen	Complete genome	MH048659	[50]
CAdV-1	NA	USA	Grizzly bear (*Ursus arctos horribilis*)	Brain	Hexon	MF621581	[103]
CAdV-2	113-3F-c01	Italy	Red fox (*Vulpes vulpes*)	Feces	E3	JX416841	[45]
CAdV-2	113-3F-c04	Italy	Red fox (*Vulpes vulpes*)	Feces	E3	JX416842	[45]
CAdV-2	18Ra-54	Korea	Raccoon dog (*Nyctereutes procyonoides*)	Isolate	Complete genome, hexon, fiber, Penton	OP644981, OP645072, OP645070, OP645074	[61]
CAdV-2	18Ra-65	Korea	Raccoon dog (*Nyctereutes procyonoides*)	Isolate	Hexon, fiber, penton	OP645073, OP645071, OP645075	[61]

CAdV-1: canine adenovirus type 1. CAdV-2: canine adenovirus type 2. NA: not available. UK: United Kingdom. USA: United States of America.

**Table 3:** Distinctive residues in the ammino acid position 388 of the deduced hexon protein of CAdV-1 reported in wild and domestic animals.

Geographic Area	Host Species	Distinctive Residue in Position 388	References
Europe	Red and arctic foxes (Norway and Svalbard)	388-aspartate (Asp)	[49]
	Dogs and wild canids (Italy and France)	388-serine (Ser)	[62,75,78]
Worldwide	Dogs (global) and gray wolves (Canada)	388-asparagine (Asn)	[72]

## Data Availability

No new data were created or analyzed in this study.
